# Higher body fatness in intrauterine growth retarded juvenile pigs is associated with lower fat and higher carbohydrate oxidation during ad libitum and restricted feeding

**DOI:** 10.1007/s00394-013-0567-x

**Published:** 2013-08-02

**Authors:** Ricarda Krueger, Michael Derno, Solvig Goers, Barbara U. Metzler-Zebeli, Gerd Nuernberg, Karen Martens, Ralf Pfuhl, Constanze Nebendahl, Annette Zeyner, Harald M. Hammon, Cornelia C. Metges

**Affiliations:** 1 Institute of Nutritional Physiology ‘Oskar Kellner’, Leibniz Institute for Farm Animal Biology (FBN), 18196 Dummerstorf, Germany; 2 Institute of Genetics and Biometry, Leibniz Institute for Farm Animal Biology (FBN), 18196 Dummerstorf, Germany; 3 Institute of Muscle Biology and Growth, Leibniz Institute for Farm Animal Biology (FBN), 18196 Dummerstorf, Germany; 4 Chair of Animal Nutrition, University of Rostock, 18059 Rostock, Germany; 5 Present Address: Clinic for Swine, University of Veterinary Medicine Vienna, 1210 Vienna, Austria; 6 Present Address: Chair for Nutritional Physiology and Animal Nutrition, Martin Luther University Halle-Wittenberg, 06120 Halle, Germany

**Keywords:** Energy expenditure, Low birth weight piglets, Fat oxidation, Food restriction, Fuel selection, Plasma NEFA

## Abstract

**Purpose:**

A thrifty energy metabolism has been suggested in intrauterine growth restricted (IUGR) offspring. We characterized energy metabolism and substrate oxidation patterns in IUGR pigs in response to food restriction (FR) and refeeding (RFD).

**Methods:**

Female pigs with low (L; 1.1 kg; *n* = 20) or normal birth weight (N; 1.5 kg; *n* = 24) were fed ad libitum after weaning. Half of L and N pigs were food restricted (R; LR, NR) from days 80 to 100 (57 % of ad libitum) and refeed from days 101 to 131, while the remaining pigs were fed ad libitum (control, C). Using indirect calorimetry, carbohydrate and fat oxidation (COX, FOX), energy expenditure (EE) and balance (EB), resting metabolic rate (RMR) [all related to kg body weight^0.62^ (BW)] and RQ were determined at 4 days before (day 76) and after (day 83) beginning of FR, 4 days before (day 97) and after (day 104) end of FR and 25 days after beginning of RFD (day 125). Body fat and muscle weights were determined at day 131.

**Results:**

In spite of higher relative food intake (FI), BW was lower in L pigs. In L pigs, physical activity was lower at age 76 and 83 days compared to N pigs. IUGR did not affect EE or RMR, but resulted in higher COX and lower FOX, causing greater and earlier onset of fat deposition. During FR, EE and RMR of R pigs dropped below that of C pigs, and BW gain was delayed by 30 % irrespective of birth weight. In response to FR, COX decreased and FOX increased. During FR, in LR pigs FOX was ~50 % of that in NR pigs. After 4 days, but not 25 days of RFD, EB and fat synthesis were higher in pigs previously subjected to FR, indicating early catch-up fat. In R pigs, BW and the abdominal fat proportion were lower at 131 days.

**Conclusions:**

Differences in food intake and substrate oxidation pattern, but not in EE and RMR, between L and N pigs were reflected in higher body fat proportions but lower body and muscle weights in L pigs. Refeeding following FR was initially associated with increased FI, a more positive EB and a more intense stimulation of fat synthesis which did not persist after 25 days of refeeding.

**Electronic supplementary material:**

The online version of this article (doi:10.1007/s00394-013-0567-x) contains supplementary material, which is available to authorized users.

## Introduction

Intrauterine growth retardation (IUGR) has been recognized as a condition which increases the propensity for adult metabolic disorders such as diabetes type 2 or obesity [1, 2]. It has been suggested that catch-up growth following IUGR is characterized by a disproportionately higher rate of fat gain and that this is in part driven by suppressed thermogenesis [3]. It has been shown that a greater weight gain and fat mass early in life after thinness at birth are risk factors for obesity [4, 5]. The pig is increasingly used as a biomedical animal model due to its similarity to human physiology [6, 7]. In pigs, 20 % of the littermates show naturally occurring IUGR leading to reduced postnatal and muscle growth and increased body fatness during puberty and young adulthood [8–13]. However, whether IUGR is associated with alterations of components of energy expenditure (EE) or substrate oxidation pattern during early postnatal life, to our knowledge, is not known.

The ability to adjust nutrient oxidation to the availability of fuels is an important mechanism for organisms to adapt to differing physiological and nutritional conditions. Temporary food restriction (FR) or starvation are associated with decreased EE and resting metabolic rate (RMR), as well as increased fat oxidation (FOX) and decreased carbohydrate oxidation (COX) [14–16]. However, it is a matter of debate whether a period of energy restriction can lead to a faster rate of fat recovery relative to lean tissue recovery during realimentation [17].

We therefore investigated in a porcine model whether IUGR individuals as compared to age-matched normal birth weight (BiW) littermates show lower EE, RMR, and FOX. We further hypothesized that FR in IUGR pigs (1) impairs their BW development and stimulates their propensity for fat deposition during refeeding (RFD), (2) stimulates food intake (FI) during RFD and (3) leads to disproportionately altered muscle weights and body composition after a period of RFD. Thus, we characterized components of energy metabolism and determined within-day changes of fuel selection in response to age, FR and RFD in normal BiW and IUGR pigs. In an attempt to link EE to the metabolic level, we also monitored the course of plasma NEFA concentration.

## Methods

### Animals and experimental design

The experimental procedures were carried out in accordance with the German Animal Protection regulations and were approved by the relevant authorities of the Land Mecklenburg-Vorpommern, Germany (Landesamt für Landwirtschaft, Lebensmittelsicherheit und Fischerei, Mecklenburg-Vorpommern; permission no. LALLF M-V/TSD/7221.3-1.1-049/09).

Seventeen German Landrace litters (2nd to 4th parity sows; mean litter size *n* = 15 ± 0.6; mean piglet weight = 1.4 ± 0.02 kg) were used. Pairs of female littermates were selected of which one piglet had a low (L; 0.8–1.2 kg; *n* = 20) and the other piglet had normal (N; 1.3–1.8 kg; *n* = 24) BiW. Low BiW was defined as having a BiW less than the lower quartile of the average litter BiW in our pig breeding facility [18]. The experiment was performed in 4 blocks of 12 pigs each, and each block contained all groups. Four pigs had to be removed from the experiment due to sudden death, illness or insufficient FI.

Piglets were suckled by their dam. Post-weaning diets were formulated to supply energy and nutrients at or above recommendations [19] (Table 1). After weaning at 28 days of age, piglets were fed ad libitum a diet formulated for the post-weaning period (‘Baby Crisb,’ Bergophor Hohburg Mineralfutter GmbH, Hohburg, Germany) plus oat flakes (Holstenmühle W. Smidt & Co. KG, Lübeck, Germany) and sucrose (Nordzucker, Braunschweig, Germany) until day 79 of age. From day 80 of age onwards, the pigs received a grower diet (‘Vormast CaFo TOP,’ Trede und von Pein GmbH, Itzehoe, Germany) plus oat flakes and sucrose (Online Resource 1; Table 1).

**Table 1:** Diet components, dry matter (DM), calculated crude nutrient composition, and metabolizable energy (ME), classified according to age of pigs

	Age (day)			
	28–41	42–79	80–130	80–100 (R pigs)
Diet components (weight % of daily food allowance)				
Starter feed^a^	50	46.5	–	–
Grower feed^b^	–	–	68.5	100 (= 825 g/day)
Oat flakes^c^	30	33.8	20.7	–
Sucrose^d^	20	19.7	10.8	–
DM (%)	98	98	98	98
Crude nutrients (g/kg)				
Crude ash	35	34	44	57
Crude protein	162	159	165	197
Crude fat	94	92	38	32
Carbohydrates	604	612	641	576
Crude fiber	16	16	39	51
Starch	314	330	440	447
Sugar	278	270	148	57
ME^e^ (MJ/kg DM)	16.9	16.9	15.2	14.7

^a^‘Baby Crisb,’ Bergophor, Hohburg Mineralfutter GmbH, Hohburg, Germany

^b^‘Vormast CaFo TOP,’ Trede und von Pein GmbH, Itzehoe, Germany; during food restriction diet was devoid of sugar and oat flakes

^c^Holstenmühle W. Smidt & Co. KG, Lübeck, Germany

^d^ Nordzucker, Braunschweig, Germany

^e^Calculated according to GfE (2008): ME (MJ/kg DM) = 0.021503 × crude protein (g/kg DM) + 0.032497 × crude fat (g/kg DM) – 0.021071 × crude fiber (g/kg DM) + 0.016309 × starch (g/kg DM) + 0.014701 × organic residue (g/kg DM)

At 79 days of age, we randomly selected half of the N and L pigs as control (C) groups which were continued on ad libitum feeding (NC, *n* = 12; LC, *n* = 10) until the age of 130 days. The remaining pigs were food restricted (R) (NR, *n* = 12; LR, *n* = 10) at a target energy intake (EI) level of 60 % of the control group for 21 days before they were returned to ad libitum feeding which was continued until the age of 130 days (Online Resource 2). Piglets were transferred to single pens (2.40 × 1.40 m; with slatted floor) to allow for individual FI measurements until the end of the experiment. Room temperature and relative humidity were 22 °C and 65 %, respectively, and light was on between 0600 and 1900 h. Pigs were fed twice daily with 50 % of their daily allowance at 0800 and 1500 h, respectively. Water was available ad libitum from nipple drinkers.

### Body weight, food intake and food analysis

The BW (kg) and FI (g/kg BW) were averaged weekly. BW, BW gain, FI and food conversion ratio (FCR) on defined days or within a period were calculated by polynomial fitting up to 9th (BW) or 10th (FI) order (Table Curve 2D, version 5.01, Systat Software GmbH, Erkrath). Food intake for R pigs was fitted separately before and after FR.

Proximate nutrient analysis of oat flakes and commercial food was performed at the Chair of Animal Nutrition, University of Rostock, according to standardized methods [20]. Dry matter was determined after 3-h drying at 105 °C (method no. 3.1). Crude fiber was analyzed by FOSS analyzer (Fibertec™ 2010, FOSS GmbH, Rellingen, Germany) and crude fat according to the Soxhlet procedure (method no. 5.1.1). Crude protein and ash were measured using Dumas combustion (vario MAX, Elementar Analysensysteme GmbH, Hanau, Germany). Sugars were analyzed gravimetrically by the Fehling method, and starch determination was based on glucose analysis by HPLC on a HPX—87 C column (BIORAD) protected with a CO_3_^−^ cartridge (BIORAD) after enzymatic degradation with α-amylase and amyloglucosidase (TERMAMYL, Novo Nordisk A/S, Denmark).

### Indirect calorimetry measurements, procedures and calculations

To determine components of energy metabolism, open-circuit indirect calorimetry was used. The chambers (1.5 m^3^) were air-conditioned to maintain a constant temperature of 22 °C at ~70 % relative humidity. The air flow through the chambers was 7 m^3^/h. Concentrations of CO_2_ and O_2_ in chamber air samples were analyzed by infrared absorption (UNOR 610, MAIHAK AG, Hamburg, Germany) and paramagnetically (OXYGOR 610, MAIHAK AG, Hamburg, Germany), respectively. Physical activity (PA) was monitored with infrared detectors (Steinel, Herzebrock-Clarholz, Germany). When the infrared beam was interrupted by the animal for 1 s, ten impulses of PA were recorded. This allowed the calculation of total PA during each stay in the calorimetric chamber.

Calorimetric measurements were performed over 23 h each at 5 consecutive time points (and ages) during the experiment: 4 days prior to FR (T1, 76 days), on day 4 (T2a, 83 days) and day 18 (T2b, 97 days) of FR, and on day 4 (T3a, 104 days) and day 25 (T3b, 125 days) of RFD (Online Resource 2). After overnight food withdrawal, calorimetric measurements were started at 0830 h and pigs were fed half of the daily feed allowance at 0930 and 1600 h. Food access was denied between 1800 h until the next morning 0800 h when measurements were terminated. Light was turned off from 1900 h until 0600 h. Pigs were weighed directly before and after measurements or biweekly when no calorimetric measurement was performed. Water was available ad libitum. Total oxygen consumption ($V_{{{\text{O}}_{2} }}$) and CO_2_ production ($V_{{{\text{CO}}_{2} }}$) were measured every 6 min corresponding to 230 values per animal in 23 h. Values were added up over 23 h and subsequently normalized to 24 h to calculate daily EE (kJ/d) and ‘net disappearance rates’ of carbohydrates (COX, g/d) and lipids (FOX, g/d) from their respective metabolic pools (where ‘disappearance’ is largely oxidation) [21]:

1 $$ {\text{COX}} = 4.12 \, \times \, V_{{{\text{CO}}_{2} }} - 2.91 \times V_{{O_{2} }} - 2.33 \, \times \, m_{\text{urinary\,N}} $$

2 $$ {\text{FOX}} = 1.69 \times V_{{{\text{O}}_{2} }} - 1.69 \times V_{{{\text{CO}}_{2} }} - 2.03 \times m_{\text{urinary\,N}} $$

3 $$ {\text{EE}} = 3.91 \times V_{{{\text{O}}_{2} }} + 1.10 \times V_{{{\text{CO}}_{2} }} - 1.93 \times m_{\text{urinary\,N}} $$

where $V_{{{\text{CO}}_{2} }}$ and $V_{{{\text{O}}_{2} }}$ (in L/d) are 24-h values and *m*_urinary N_ (g/d) refers to the estimated urinary N excreted on the measurement day. Urinary N excretion was estimated based on published values measured under similar experimental conditions as in the present study [15, 22–24]. Nitrogen excreted via urine corresponded to 40 % of ingested N, whereas during the RFD period (T3a and T3b; R pigs), it was estimated to be equivalent to 35 % of ingested N. Estimated daily urinary N excretion served as basis to calculate protein disappearance rate (P_dis_), expressing the difference between N intake and urinary N excretion (*m*_urinary N_) [21]:

4 $$ {\text{P}}_{\text{dis}} = 6.25 \times m_{\text{urinary\,N}} $$

The daily respiratory quotient (RQ) was calculated as 24-h production of CO_2_ (L) divided by 24-h consumption of O_2_ (L). Resting metabolic rate (RMR) as a measure of basal metabolic rate (or fasting heat production) was derived from the 10 lowest EE values over the 23-h measurement period that were averaged and normalized to 24 h, reflecting energy metabolism due to basal metabolic rate and not due to FI, digestion of nutrients or PA. Energy expenditure due to PA (EE_PA_) was calculated according to 0.9 × 24-h EE, 24-h RMR, assuming that the thermic effect of food is ~10 % of EE [25, 26]. Energy balance (EB) was calculated by subtracting 24-h EE from daily energy intake (EI) in MJ ME. Daily COX, FOX, EE, EB and RMR were then expressed relative to metabolic BW (kg BW^0.62^) and COX additionally to the amount of FI (g/kg BW^0.62^ kg FI^−1^). As an indicator for efficiency of energy utilization, the *Q* value was calculated for individual N and L pigs during ad libitum feeding as 24-h EE (MJ ME) divided by corresponding daily energy intake (MJ ME). The higher the *Q* value, the higher the relative heat loss and the less energy was retained as body mass (protein accretion and/or fat deposition).

### Diurnal substrate oxidation pattern and plasma NEFA concentrations

Individual amounts of COX and FOX were calculated for 6-min intervals [21]. Because P_dis_ was calculated per d based on literature values, it was divided in 6-min intervals. Then, COX, FOX and P_dis_ values were expressed as g/kg BW^0.62^ (6 min)^−1^ and transferred to the corresponding energetic values of carbohydrates (17.2 kJ/g), fat (38.9 kJ/g) and protein (17.8 kJ/g), respectively [27]. The 6-min energetic values of the 3 macronutrients were added up to total EE_6 min_ [kJ/kg BW^0.62^ (6 min)^−1^]. Subsequently, the % contribution of each macronutrient was derived and plotted against time of the day. A proportion of FOX < 0 % of total EE indicates fat deposition.

In a further experimental subset, 33 piglets were allocated to the 4 experimental groups as described above to determine plasma NEFA concentrations at day 21 of FR (C and R pigs), day 7 (R pigs only) and day 21 of RFD (C and R pigs). To link diurnal FOX data with plasma NEFA concentrations, the plasma sampling times were expressed relative to the feeding times of the respiration experiment. Relative to the morning feeding blood samples were collected in Fluoride-EDTA coated tubes (1.2 mg EDTA/ml blood; Monovette, Sarstedt, Nümbrecht, Germany) at -0.1, 1, 2, 3, 4, 5, 6, 7, 8 and 12 h. Blood was put on ice and subsequently centrifuged 20 min at 1,573×*g* and 4 °C (Heraeus Multifuge 3 L-R, Kendro Laboratory Products, Osterode, Germany) to isolate the plasma, before storage at −80 °C until analysis. Plasma NEFA concentrations were analyzed using kit no. 600-215S from Wako Chemicals GmbH (Neuss, Germany).

### Body composition

Nine randomly selected pigs per group were slaughtered after an overnight fast at 131 (±1) d of age (after 30 days of RFD) by electro-stunning followed by exsanguination in the experimental abattoir of the Institute. Subsequently, liver, left and right kidney, pancreas and heart, as well as peritoneal and omental fat (both together termed abdominal fat) were removed and weighed. Twenty-four hours after slaughter several skeletal muscles (*m. longissimus dorsi*, *m. semitendinosus*, and *m. semimembranosus*), backfat and subcutaneous fat covering neck, shoulder and ham were dissected from the left carcass half and weighed.

### Statistical analysis

Data were analyzed by the MIXED procedure of SAS (version 9.3; SAS Inst. Inc., Cary, NC, USA). Post hoc comparisons were performed using the Tukey–Kramer test. Significance was considered at *P* ≤ 0.05, and trends were discussed at 0.05 < *P* ≤ 0.1. Results are reported as least square means (LSM) ± SE. During model development, the original model included ‘block’ as a factor but because it had no significant influence as main effect or interaction it was excluded from the statistical model. To evaluate the variables COX, FOX and EE, and COX, FOX and P_dis_ as % of EE, EB, EE_PA_, RQ, *Q* value during ad libitum feeding, RMR and PA, the model included the fixed factors BiW class (N, L), feeding type (C, R), the repeated factor time point (T1, T2a, T2b, T3a, T3b) and interactions, and the sow as random factor. The latter allows us to model the dependency of the littermate piglets from the same sow. Food intake, food conversion ratio (FCR) and BW were analyzed separately at days 28, 79, 90, 101, 129/131, considering the fixed factors BiW class (N, L), feeding type (C, R) and interactions and the sow as random factor. The time course of plasma NEFA concentrations at day 21 of FR and on day 7 and day 21 of RFD was compared between groups at each time point separately using a model with the fixed factors BiW class (N, L), feeding type (C, R) and interactions with the sow as random factor.

## Results

### Food Intake, body weight and composition and food efficiency

Absolute food intake was lower in L pigs until age 79 days (*P* < 0.05; Table 2). However, relative FI/kg BW was higher in L as compared to N pigs over the whole experimental period (*P* < 0.05; Table 2; Online Resource 3). Starch and sugar as well as fat intake/kg BW were affected by BiW (*P* < 0.05), time point (T1–T3) (*P* < 0.001) and feeding type (C or R) × time point interaction (*P* < 0.001), whereas sugar intake was additionally affected by feeding type (*P* < 0.001). The latter is due to the fact that FR diet was devoid of added sugar (Table 1). Overall starch and sugar intake/kg BW, as well as fat intake/kg (1.80 vs. 1.66 g/kg BW) were generally higher in L pigs than in N pigs (*P* < 0.05). At T1, BW-related fat intake was about three times as high as compared to the remaining time points (T2a–T3b) due to the higher fat content in the diet until age 79 days (Table 1).

**Table 2:** Body weight (BW), food intake (FI), and food conversion ratio (FCR) in low (L) and normal (N) birth weight (BiW) pigs food restricted for 21 days (FR; age 80–100 day) and subsequently refed (RFD) ad libitum (R), and in pigs fed ad libitum throughout the experimental period (C)

	N		L		*P* value^e^	
	C	R	C	R	BiW	Feeding type
BW (kg) at age						
1 day; birth	1.5^b^ ± 0.02		1.1^a^ ± 0.03		<0.001	
28 days; weaning	9.0^b^ ± 0.26		6.9^a^ ± 0.27		<0.001	
79 days; prior to FR	29.6^b^ ± 0.60		24.9^a^ ± 0.66		<0.001	
90 days; during FR	36^c^ ± 1.0	34^bc^ ± 1.0	31^ab^ ± 1.1	29^a^ ± 1.1	<0.001	0.070
101 days; begin RFD	43^c^ ± 1.1	39^bc^ ± 1.1	38^ab^ ± 1.2	34^a^ ± 1.2	<0.001	0.003
131 days; end of RFD^f^	71^b^ ± 1.8	69^ab^ ± 1.8	67^ab^ ± 2.0	62^a^ ± 2.0	0.012	0.061
Absolute FI (g/d) at age						
28 days; weaning	306^b^ ± 9		270^a^ ± 9		<0.001	
79 days; prior to FR	1307^b^ ± 55		1163^a^ ± 60		0.021	
90 days; during FR	1448^b^ ± 35	825^a^ ± 35	1397^b^ ± 38	825^a^ ± 38	0.486	<0.001
101 days; begin RFD	1653 ± 47	1690 ± 47	1645 ± 51	1510 ± 51	0.056	0.321
130 days; end of RFD	2521 ± 77	2569 ± 77	2518 ± 90	2546 ± 80	0.870	0.644
FI related to BW (g/kg BW)						
28 days; weaning	34^a^ ± 2.0		41^b^ ± 2.2		0.006	
79 days; before FR	44 ± 2.1		47 ± 2.3		0.162	
90 days; during FR	40^c^ ± 1.0	24^a^ ± 1.0	45^d^ ± 1.1	29^b^ ± 1.1	<0.001	<0.001
101 days; begin RFD	39^a^ ± 1.0	43^b^ ± 1.0	43^b^ ± 1.1	44^b^ ± 1.1	0.032	0.012
130 days; end of RFD	37^a^ ± 1.2	40^ab^ ± 1.2	40^ab^ ± 1.4	41^b^ ± 1.3	0.038	0.043
FCR (kg FI/kg BW gain)						
28–79 days; prior to FR	2.04 ± 0.05		1.97 ± 0.05		0.334	
80–90 days; during early FR	2.50^b^ ± 0.09	2.23^ab^ ± 0.09	2.12^a^ ± 0.10	2.12^a^ ± 0.10	0.008	0.162
91–100 days; during late FR	2.46^b^ ± 0.06	1.67^a^ ± 0.06	2.24^b^ ± 0.07	1.67^a^ ± 0.07	0.073	<0.001
101–130 days; during RFD	2.43 ± 0.07	2.40 ± 0.07	2.31 ± 0.08	2.23 ± 0.08	0.075	0.459

Values are LSM ± SE (*n* = 9–12/group)

^a–d^Within a row, LS means without a common superscript differ (*P* ≤ 0.05, Tukey–Kramer test)

^e^A trend for BiW x Feeding type interaction was observed for FI/kg BW on day 101 (*P* = 0.078) and FCR between days 91 and 100 (*P* = 0.092); BiW × Feeding type interaction effects for all other variables were not significant (*P* > 0.1)

^f^Slaughter weight

In L pigs, BW was permanently lower, compared to N pigs (*P* < 0.05; Table 2, Online Resources 4 and 5). From birth to weaning, average BW gain in N pigs was 7.5 kg and in L pigs 5.8 kg (*P* < 0.001). Also from weaning to the beginning of FR, the BW gain in N and L pigs differed, with N pigs gaining 21 kg and L pigs 18 kg, respectively (*P* = 0.004). In R pigs, relative starch and sugar intake were lower during the FR period (13 vs. 21 g/kg BW; *P* < 0.001), but higher during early RFD (T3a; 23.5 vs. 19.9 g/kg BW; *P* < 0.001). Relative fat intake during FR was ~60 % of ad libitum fed C pigs (*P* < 0.001) as targeted, with no difference between L and N pigs (*P* > 0.6). In previously food restricted N pigs, relative fat intake was higher during early RFD (T3a) (1.5 vs. 1.2 g/kg BW; *P* < 0.05). The 3-week FR delayed BW gain by about 30 % as compared to age-matched C pigs (R pigs 9.5 kg; C pigs 13.6 kg; *P* < 0.001), with no differences regarding the BiW class (Table 2). After 30 days of RFD, BW tended to be still decreased in previously food restricted pigs (*P* = 0.06), although FI/kg BW was permanently higher during RFD (*P* < 0.04; Online Resource 3).

A low BiW improved or tended to improve FCR during FR and RFD (Table 2; Online Resource 5). Furthermore, pigs during late FR showed a better FCR as well (*P* < 0.001). Irrespective of BiW and in agreement to FCR data, the *Q* value as another indicator for food utilization was lower in R compared to C pigs at the age of 104 days (Table 3; T3a; *P* < 0.001). This indicates lower heat loss in R pigs during early RFD favoring energy deposition during that period. Averaged over the total of 30 days RFD period, however, FCR of previously food restricted pigs did not differ from age-matched controls (Table 2). Irrespective of the FR and RFD periods, at age day 131, L pigs showed decreased weights of *m. semimembranosus* and *m. semitendinosus* (*P* < 0.05), higher percentage of abdominal fat (1.7 vs. 1.45 % BW; *P* = 0.002) and a trend toward an increased carcass fat proportion (5.4 vs. 5.0 % of half carcass; *P* = 0.06) (Table 4). Food restriction resulted in persistently lower weights of abdominal fat depots after 30 days of RFD independent of the pigs’ BiW (*P* = 0.015).

**Table 3:** Body weights (BW) and components of 24-h energy expenditure (EE) before (T1), during (T2a and T2b) and after (T3a and T3b) food restriction in low (L) and normal (N) birth weight pigs food restricted for 21 days (age 80–100 day) and subsequently refed ad libitum (R), and in pigs fed ad libitum throughout the experimental period (C)

	T1/76 day				T2a/83 day				T2b/97 day				T3a/104 day				T3b/125 day			
	N		L		N		L		N		L		N		L		N		L	
	C	R	C	R	C	R	C	R	C	R	C	R	C	R	C	R	C	R	C	R
BW (kg)	28.0^Aab^ ±0.9	28.3^Ab^ ±0.9	23.7^Aab^ ±1.0	23.3^Aa^ ±1.0	32.0^Bb^ ±0.9	31.8^Bb^ ±0.9	27.2^Bab^ ±1.0	26.5^Ba^ ±1.0	41.8^Cb^ ±1.0	37.2^Cab^ ±1.1	37.0^Cab^ ±1.1	32.3^Ca^ ±1.2	47.2^Db^ ±1.1	43.1^Dab^ ±1.2	42.7^Dab^ ±1.3	37.5 ^Da^ ±1.3	67.6^Eb^ ±1.6	64.5^Eab^ ±1.7	62.7^Eab^ ±1.8	57.5^Ea^ ±1.9
EI^F^ (kJ/kg BW^0.62^ day^−1^)	2370 ±131	2359^B^ ±131	2287 ±143	2388^B^ ±143	1991^b^ ±75	1390^Aa^ ±76	2051^b^ ±79	1522^Aa^ ±83	1887^b^ ±73	1264^Aa^ ±73	2129^b^ ±77	1383^Aa^ ±80	2047 ±87	2443^B^ ±88	2207 ±96	2386^B^ ±96	2217 ±127	2522^B^ ±128	2254 ±140	2515^B^ ±140
EE (kJ/kg BW^0.62^ day^−1^)	1278 ±26	1295^C^ ±26	1256 ±29	1257^B^ ±30	1248 ±25	1157^B^ ±24	1224 ±25	1129^A^ ±28	1266^b^ ±20	1059^Aa^ ±22	1299^b^ ±21	1079^Aa^ ±22	1265 ±22	1290^C^ ±22	1299 ±25	1280^B^ ±24	1288 ±32	1340^C^ ±30	1309 ±34	1373^B^ ±34
EB (kJ/kg BW^0.62^ day^−1^)	1032 ±149	1105^B^ ±150	1061 ±167	1077^B^ ±172	595^ab^ ±80	239^Aa^ ±78	787^b^ ±82	303^Aab^ ±91	629^b^ ±72	192^Aa^ ±80	780^b^ ±76	304^Aab^ ±80	701^a^ ±72	1153^Bb^ ±71	908^ab^ ±80	1106^Bb^ ±78	1022 ±105	1182^B^ ±99	977 ±113	1169^B^ ±113
RMR (kJ/kg BW^0.62^ day^−1^)	947 ±23	933^BC^ ±23	906 ±26	914^AB^ ±27	970^b^ ±19	862^Ba^ ±19	933^ab^ ±20	852^Aa^ ±22	976^b^ ±15	786^Aa^ ±17	980^b^ ±16	824^Aa^ ±17	957 ±18	980^C^ ±18	967 ±20	961^B^ ±19	968 ±21	1005^C^ ±20	997 ±23	1006^B^ ±23
RQ	0.95^A^ ±0.01	0.95^A^ ±0.01	0.94^A^ ±0.02	0.98^A^ ±0.02	0.94^Aab^ ±0.01	0.91^Aa^ ±0.01	0.99^ABb^ ±0.01	0.93^Aa^ ±0.01	0.95^Aab^ ±0.01	0.92^Aa^ ±0.01	0.99^ABb^ ±0.01	0.93^Aab^ ±0.01	0.98^ABa^ ±0.01	1.07^Bb^ ±0.01	1.03^Bab^ ±0.01	1.07^Bb^ ±0.01	1.03^B^ ±0.01	1.07^B^ ±0.01	1.04^B^ ±0.01	1.07^B^ ±0.01
*Q* value (MJ EE d^−1^/MJ EI d^−1^)	0.58^AB^ ±0.03	0.55 ±0.03	0.57 ±0.04	0.55 ±0.04	0.69^AB^ ±0.03	–	0.63 ±0.05	–	0.68^AB^ ±0.04	–	0.65 ±0.04	–	0.65^Bb^ ±0.02	0.53^a^ ±0.02	0.59^ab^ ±0.02	0.54^a^ ±0.02	0.57^A^ ±0.02	0.54 ±0.02	0.59 ±0.02	0.55 ±0.02
FOX (g/kg BW^0.62^ day^−1^)	2.47^B^ ±1.58	1.46^B^ ±1.59	2.34^B^ ±1.78	−0.88^B^ ±1.81	3.42^Bb^ ±1.03	7.05^Cb^ ±1.00	−2.05^ABa^ ±1.05	4.43^Bb^ ±1.18	2.51^Bab^ ±1.19	4.75^BCb^ ±1.34	−2.79^ABa^ ±1.25	3.96^Bb^ ±1.31	−0.84^Bb^ ±1.35	−10.1^Aa^ ±1.31	−6.57^Aab^ ±1.49	−9.92^Aa^ ±1.44	−7.05^A^ ±1.64	−11.21^A^ ±1.53	−8.37^A^ ±1.75	−12.06^A^ ±1.75
COX (g/kg BW^0.62^ day^−1^)	58.8^A^ ±3.3	61.8^B^ ±3.4	58.5^A^ ±3.8	65.3^B^ ±3.8	55.8^Aab^ ±2.7	43.1^Aa^ ±2.6	62.9^Ab^ ±2.7	46.9^Aa^ ±3.1	58.4^Aab^ ±3.0	43.1^Aa^ ±3.4	69.5^ABb^ ±3.2	45.3^Aa^ ±3.3	65.8^ABa^ ±3.2	87.6^Cb^ ±3.1	79.1^Bab^ ±3.5	86.8^Cb^ ±3.4	80.8^B^ ±4.6	92.6^C^ ±4.2	83.5^B^ ±4.9	96.5^C^ ±4.9
COX (g/kg BW^0.62^ kg FI^−1^ day^−1^)	53.9^Ba^ ±1.9	54.6^Ba^ ±1.9	60.3^Cab^ ±2.1	65.7^Cb^ ±2.1	54.3^B^ ±3.6	52.5^AB^ ±3.4	57.3^BC^ ±3.6	58.6^ABC^ ±4.1	46.4^AB^ ±2.0	52.8^B^ ±2.2	50.9^BC^ ±2.1	55.3^AB^ ±4.1	46.4^Ba^ ±1.4	52.3^Bab^ ±1.4	50.7^Bab^ ±1.6	58.0^BCb^ ±1.5	38.4^Aa^ ±1.6	41.7^Aab^ ±1.5	41.3^Aab^ ±1.7	46.5^Ab^ ±1.7

Values are 24-h LSM ± SE (*n* = 7–12/group), *P* values are provided in Online Resource 5

^ab^Within a row, LSM without a common lower case superscript differ among the groups within one time point (*P* ≤ 0.05, Tukey–Kramer test)

^A–E^Within a row, LSM without a common capital superscript differ within one group between the time points (*P* ≤ 0.05, Tukey–Kramer test)

^F^EI, energy intake; EB, energy balance; RMR, resting metabolic rate; RQ, respiratory quotient; FOX, fat oxidation; COX, carbohydrate oxidation

**Table 4:** Weights of organs, fat depots and skeletal muscles at the age of 131 days in low (L) and normal (N) birth weight (BiW) pigs food restricted for 21 days (age 80–100 day) and subsequently refed ad libitum (R), and in pigs fed ad libitum throughout the experimental period (C)

	N		L		*P* value^c^	
	C	R	C	R	BiW	Feeding type
Fat depots						
Omental fat	508^ab^ ± 28	450^a^ ± 28	567^b^ ± 30	495^ab^ ± 28	0.077	0.044
Ventral visceral fat	550^ab^ ± 48	493^ab^ ± 48	671^b^ ± 52	453^a^ ± 48	0.432	0.016
Σ Abdominal fat	1058^ab^ ± 66	943^a^ ± 66	1237^b^ ± 71	970^ab^ ± 66	0.150	0.015
Subcutaneous fat^d^ covering						
Shoulder	400 ± 37	445 ± 37	393 ± 39	360 ± 37	0.171	0.893
Neck	335 ± 32	326 ± 32	373 ± 34	350 ± 32	0.631	0.162
Ham	890 ± 64	868 ± 64	960 ± 69	749 ± 64	0.712	0.104
Backfat^d^	843 ± 87	755 ± 87	874 ± 94	676 ± 87	0.789	0.137
Σ Fat depots^e^	3527 ± 227	3337 ± 227	3833 ± 244	3083 ± 227	0.912	0.065
Organs						
Liver	1240 ± 50	1295 ± 50	1284 ± 53	1241 ± 50	0.911	0.909
Kidneys (mean)	132 ± 5	134 ± 5	131 ± 5	129 ± 5	0.471	0.993
Heart	283 ± 11	274 ± 11	270 ± 12	254 ± 11	0.178	0.311
Pancreas	121 ± 7	117 ± 7	118 ± 7	109 ± 7	0.450	0.348
Muscles^d^						
*M. longissimus dorsi*	1677 ± 89	1727 ± 89	1670 ± 95	1493 ± 89	0.203	0.494
*M. semimembranosus*	1038 ± 42	1011 ± 42	947 ± 45	875 ± 42	0.020	0.275
*M. semitendinosus*	332^b^ ± 13	298^ab^ ± 13	303^ab^ ± 14	262^a^ ± 13	0.035	0.018

Values are LSM ± SE (*n* = 8–9/group); all weights are in g

^ab^Within a row, LSM without a common superscript differ (*P* ≤ 0.05, Tukey–Kramer test)

^c^BiW × Feeding type interaction effects for all variables were not significant (*P* > 0.1)

^d^Tissue weights are related to the left half carcass

^e^Sum of the weights of abdominal fat depots, subcutaneous fat covering shoulder, neck and ham, and backfat

### 24-h energy metabolism

#### Effects of birth weight and age

Birth weight did not affect EE, EB and RMR (all *P* > 0.3; Table 3; Online Resource 5). However, PA was lower in L pigs at T1 (3.0 vs. 4.1 h/day) and T2a (3.3 vs. 4.4 h/day) (*P* = 0.040), but EE_PA_ was not affected by BiW (*P* > 0.4).

With increasing age, the contribution of FOX to EE steadily decreased in ad libitum fed pigs with normal BiW (Table 3; FOX became negative). However, FOX was affected by BiW (*P* = 0.006; Online Resource 5). At age 83 days (T2a), but not at 76 days (T1), FOX was lower in L compared to N pigs (*P* = 0.007), in spite of similar fat intake in the two groups. In LC pigs, FOX was already negative at the age of 83 days (T2a), whereas in NC pigs, FOX became negative later, on day 104 (T3a; Table 3). Irrespective of age and feeding level, overall, L pigs generally exhibited a lower FOX (i.e., greater fat deposition) throughout the experimental period compared to N pigs (N vs. L: −0.8 vs. −3.2 g/kg BW^0.62^; *P* = 0.006). In line with this, L pigs showed a higher RQ value (N vs. L: 0.98 vs. 1.00; *P* = 0.006; Table 3). In contrast, a low BiW was associated with a higher COX, compared to N pigs (N vs. L: 65 vs. 69 g/kg BW^0.62^; *P* = 0.026; Table 3).

#### Effects of food restriction and refeeding

**Fig. 1:**
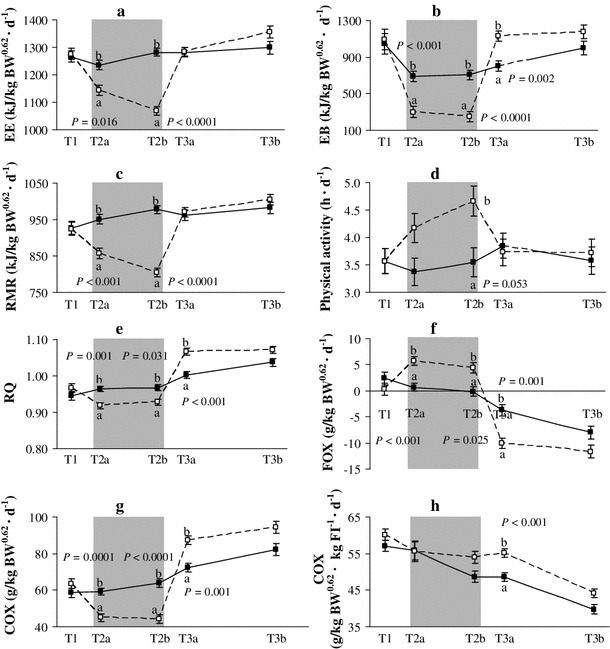
Energy expenditure (EE) (**a**), energy balance (EB) (**b**), resting metabolic rate (RMR) (**c**), physical activity (**d**), RQ (**e**), fat oxidation (FOX) (**f**) and carbohydrate oxidation (COX) (**g**, **h**) before (T1), during (T2a and T2b) and after (T3a and T3b) food restriction (pooled LSM ± SE) for ad libitum fed pigs (*solid line with filled square*) and food restricted pigs (*dashed line with open square* ; *n* = 7–12/group). The food restriction period is *shaded in gray*. *FI* food intake

Energy expenditure and RMR decreased in response to FR in R pigs as compared to age-matched C pigs with ad libitum feeding (Fig. 1; T2a and T2b: both *P* < 0.001), also resulting in a less positive EB irrespective of the pigs’ BiW (Table 3). Resting metabolic rate was significantly reduced only after 17 days of FR (T2b), and at the same time, PA in R pigs was higher compared to C pigs (*P* = 0.024). However, EE_PA_ was similar between R and C pigs *(P* = 0.514). Food restriction increased FOX when related to BW and expressed as a percentage of total EE (*P* = 0.008), which was irrespective of BiW (Table 3; Online Resource 5). During FR COX related to BW and as a percentage of total EE decreased (Fig. 1; T2a and T2b). Four d after the start of RFD (day 104; T3a), EI tended to be higher in R than in C pigs (*P* = 0.07), resulting in a more positive EB as compared to C pigs (*P* = 0.002; Table 3; Fig. 1). The more positive EB only after 4 days of RFD (T3a) in R pigs was associated with a distinctly decreased FOX which went below the level found in C pigs (Table 3). In addition, at 4 days of RFD (T3a), COX adjusted for BW and FI was higher in R than in C pigs (C vs. R, 48.6 vs. 55.2 g/kg BW^0.62^FI^−1^; *P* < 0.001), irrespective of BiW (Fig. 1).

### Substrate oxidation pattern during 24 h and plasma NEFA concentrations

**Fig. 2:**
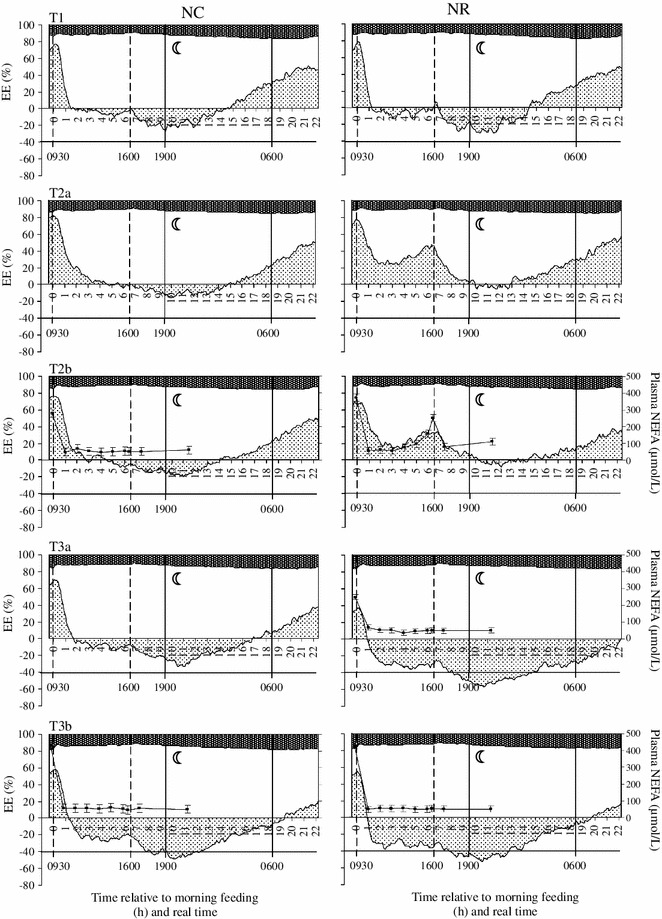
Diurnal patterns of fat, carbohydrate and protein disappearance (from *bottom* to *top*: fat oxidation, *gray shading*; carbohydrate oxidation, *blank area*; protein disappearance, *dark shading*) expressed as % of total energy expenditure (EE; *left y-axis*; LSM; SE were omitted for the sake of clarity) and related to plasma NEFA concentration (*right y-axis*, *solid line with filled square*; LSM ± SE) in ad libitum fed pigs with normal birth weight (NC; *left panels*; *n* = 6–7/group) and food restricted pigs with normal birth weight (NR; *right panels*; *n* = 9/group) before (*top panel*: T1), during (*middle panels*: T2a and T2b) and after (*bottom panels*: T3a and T3b) food restriction. *Vertical dashed lines* indicate the feeding time points within day. The time period between 1900 and 0600 h marked with a *half-moon* represents the night phase. *Negative fat oxidation values* indicate fat synthesis

**Fig. 3:**
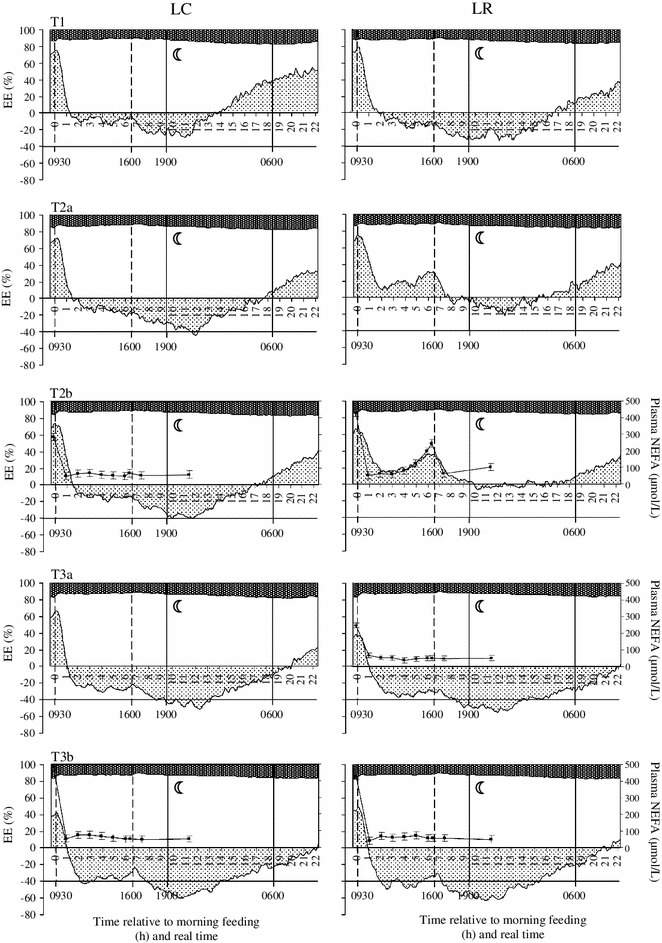
Diurnal patterns of fat, carbohydrate and protein disappearance (from *bottom* to *top*: fat oxidation, *gray shading*; carbohydrate oxidation, *blank area*; protein disappearance, *dark shading*) expressed as % of total energy expenditure (EE; *left y-axis*; LSM; SE were omitted for the sake of clarity) and related to plasma NEFA concentration (*right y-axis*, *solid line with filled square*; LSM ± SE) in ad libitum fed pigs with low birth weight (LC; *left panels*; *n* = 8/group) and food restricted pigs with low birth weight (LR; *right panels*; n = 8–9/group) before (*top panel*: T1), during (*middle panels*: T2a and T2b) and after (*bottom panels*: T3a and T3b) food restriction. *Vertical dashed lines* indicate the feeding time points within day. The time period between 1900 and 0600 h marked with a *half-moon represents* the night phase. *Negative fat oxidation values* indicate fat synthesis

Although substrate oxidation pattern (COX and FOX in  % of total EE) varied in response to age (both *P* < 0.0001) and feeding type (at T2a to T3a: COX, *P* < 0.005; FOX, *P* < 0.008), carbohydrates were the primary energy source (>78 % of EE; Figs. 2 and 3) and their contribution to total EE was higher in L than in N pigs (*P* = 0.005). In contrast, in L pigs FOX % was lower (*P* = 0.006).

Independent of age, the intake of the morning meal after an overnight fast caused a sharp decrease in FOX, followed by a transiently neutral fat balance for 1.5–2 h in NC pigs (Fig. 3). Simultaneously, and in parallel with the course of FOX, plasma NEFA concentrations dropped within 1 h after provision of food and remained at a low level over the next 12 h. In N pigs, FR (T2a and T2b) considerably increased FOX suggesting fat synthesis to not occur during the day. After the afternoon meal, FOX declined and increased again 6 h later to continue until the next morning (Figs. 2 and 3). In R pigs, the plasma NEFA concentrations started to re-increase 4–5 h after the intake of the morning meal and were higher than in C pigs (*P* < 0.001; Figs. 2 and 3; Online Resource 6). Additionally, in R pigs, plasma NEFA concentrations were higher prior to the morning meal than prior to the afternoon meal (402 and 248 μmol/L, *P* < 0.001), respectively, which is due to the longer time without food between afternoon and morning meal (18 h). In contrast, in C pigs, plasma NEFA concentrations from 2 h after the meal onwards remained at the same low level (47–70 μmol/L; Figs. 2 and 3; Online Resource 6). In LR pigs, after 4 days of FR (T2a), 1.5 h after the afternoon meal fat balance became neutral before FOX re-increased 9 h after the meal (Fig. 3). In contrast, in age-matched NR (T2a) pigs, FOX increased already 6 h after the afternoon meal. However, 24-h FOX was not different (LR vs. NR, T2a, *P* = 0.96; Figs. 2 and 3).

Between day 104 (T3a) and day 125 (T3b) of age, the NC pigs rapidly entered a phase of elevated endogenous fat synthesis (i.e., negative FOX) after the morning meal. This was enhanced by the consumption of the afternoon meal and lasted 9–12 h after that, even after withdrawal of food (1800 h). At the younger ages (T1 to T2b), the switch from fat synthesis to FOX during the night occurred earlier than at the later stages. Higher fat synthesis (i.e., negative FOX) was balanced by increased COX. In general, plasma NEFA values did not differ between N and L pigs (*P* > 0.1). However, in ad libitum fed N and L pigs plasma NEFA levels prior to the morning meal increased with age and were nearly twice as high at 125 days (T3b) than those observed 3 weeks earlier (T2b) (Online Resource 6). After 4 days of RFD (T3a) in R pigs, FOX only briefly occurred previous to the morning meal (1 h; Figs. 2 and 3). As reflected by a RQ value of 1.07 after 4 days of RFD (T3a), 24-h fat synthesis overcompensated in R pigs as compared to age-matched C pigs (Table 3). At day 125 (T3b), the substrate oxidation pattern was similar in all groups.

## Discussion

### Effects of birth weight

In spite of the higher BW-related FI in L pigs throughout the experimental period (with exception of day 79), BW and BW gain were lower in L pigs. Also, selected muscle weights were lower, but body fatness was still higher even after FR and RFD. Others also have found that IUGR was associated with impaired postnatal growth, lower lean mass, a higher fat percentage and intramuscular fat, enlarged, but lower numbers of muscle fibers, as well as a higher proportion of connective tissue [10, 11, 28–31]. It might be speculated that the lower muscle mass of IUGR pigs is in part responsible for their higher body fatness because it translates into less energy-consuming metabolically active tissue. As a result, more energy might be available to be deposited as fat [10, 32]. Similar observations have been made in human subjects previously small for gestational age at birth, and formerly malnourished children and adults where the body fat content was higher than in well-nourished individuals at the same age [32]. This was termed the ‘catch-up fat phenomenon,’ whereas the rebuilding of muscles was delayed [32]. It has been reported previously that the development of the gastrointestinal tract of IUGR pigs and the maturation of the digestive function post-weaning were delayed [13, 33, 34]. This is thought to contribute to a reduced efficiency of nutrient utilization during growth [8]. However, in the present study, a low BiW improved or tended to improve FCR during FR and RFD. Other reports are contradictory in regard to increased energetic efficiency during FR as compared to ad libitum feeding in juvenile pigs [15, 35]. An increased food or energetic efficiency might suggest that FR and IUGR have induced a thrifty metabolism. During FR, this was also reflected by a reduced basal metabolic rate and a positive EB, which still allowed some BW gain.

In contrast to our hypothesis, we could not find differences in EE and basal metabolic rate between L (IUGR) and N pigs. However, PA was lower in L pigs at T1 and T2a, which might have contributed to greater fat deposition. Our data suggest that in ad libitum fed L pigs fat deposition begins at an earlier age than in N pigs which did not show a negative FOX until 20 days later on day 104. Irrespective of age and feeding level, L pigs generally exhibited a lower FOX (i.e., greater fat deposition) throughout the experimental period, although their relative fat intake was ~10 % higher, which should have rather stimulated FOX [36]. A higher RQ value in L pigs confirms higher fat synthesis. Lower FOX contrasted with a higher COX which might be a consequence of the ~10 % higher relative food and carbohydrate intake in L pigs and related to the higher propensity for fat deposition, as glucose is used as a precursor for fatty acid synthesis and to supply NADPH for fatty acid synthesis. Thus, the difference in fuel selection of L pigs as compared to N pigs underlies the propensity for greater fat deposition in IUGR individuals as observed in this study as well as by others [10, 37, 38].

### Effects of food restriction and refeeding

Temporary FR, dieting or starvation, is associated with decreased EE and basal metabolic rate, as well as increased FOX and decreased COX [14–16, 39] which was confirmed by the present data. That the feeding level affects maintenance requirements has been recently reported in growing pigs [40]. The decrease in BW-adjusted RMR and EE is considered a mechanisms to survive deficient energy supply and to oppose FR-induced BW loss [41, 42]. Koong et al. [43, 44] showed in pigs that the fasting heat production was highly correlated with the mass of metabolically active organs and suggested that the mass of viscera is critical in the determination of RMR. In rats and humans gut and liver contribute 30–40 % to resting EE [42]. That RMR was significantly reduced only after 17 days of FR, indicates that it required ~2 week to adjust the visceral organs to the new feeding level. Similar to food restricted mice [45], PA in R pigs was higher compared to C pigs during FR, possibly due to food searching behavior. In line with another study [15], however, EE_PA_ was unrelated to FR probably resulting from proportional similar changes of RMR and EE (Table 3). The EE_PA_ amounted to 14 % of total EE which is similar to values reported earlier in pigs (8–18 %) [15, 46].

Decreased EE during FR and a re-increase due to RFD were found earlier in pigs [15]. In adult human weight regainers, a reduced resting EE after 6 months of realimentation was observed [47]. In contrast, 30 days of RFD was not long enough to have our R pigs return to the BW of their ad libitum fed age-matched controls. Similarly, in growing pigs (BW 52 kg), 7 days of FR (60 % of ad libitum FI) followed by 1 week of RFD resulted in reduced BW gain and lower BW (71 vs. 74 kg) [15]. Seventy days-old pigs fed restrictively for 28 to 60 days (until age 98 or 130 days) and subsequently refed ad libitum until the age of 140 days, did also not compensate the BW difference to ad libitum fed pigs [29]. In contrast, 62 days of 60 % of ad libitum FI in pigs starting at weaning (day 28) and subsequent RFD did not result in BW differences at the age of 145 days compared to the respective controls [30]. Thus, the extent of BW reduction after FR and ad libitum RFD is a function of age (i.e., growth rate), degree and duration of FR and RFD.

The intake of fat in R pigs during FR was 26.4 g/d (825 g food/d with 3.2 % crude fat). In early FR (T2a), we found that in NR pigs ~60 g fat/d was oxidized. This suggests that in NR pigs, more than 50 % of total FOX must be accounted for by fat depot mobilization and/or an increase in endogenous fat synthesis. We found higher plasma NEFA concentrations in R pigs pointing to an increased fat depot mobilization (Figs. 2 and 3; Online Resource 6). That FR is associated with endogenous fat synthesis was found in another indirect calorimetry study which was supported by tracer data showing endogenous fatty acid synthesis in food restricted mice (70 % of ad libitum intake) [16]. In contrast, in ad libitum fed NC pigs, the dietary fat intake at T2a was 54 g/day and the measured FOX amounted to 26 g/day suggesting that >50 % of the consumed fat is deposited in the body. The oxidized amount of fat in LR pigs (~30 g fat/day) was similar to what was ingested with the food and suggests that fat balance in L pigs was neutral during the FR period. In contrast, the fat balance of the age-matched LC pigs was + 35 g/day (fat intake 53 g/d; FOX 17.7 g/day). Thus, NR and LR pigs oxidized 2.3 and 1.7 times as much fat, respectively, than their age-matched, ad libitum fed counterparts. In addition, in LR pigs, FOX was only ~50 % of what was oxidized in NR pigs during the early FR period. This pointed to less fat mobilization in L pigs during FR. During the same period, COX (% of EE) was higher in L compared to N pigs, suggesting a higher glucose utilization to supply NADPH for fatty acid synthesis and as a precursor for fat synthesis [16, 48, 49].

The positive EB during early RFD was accompanied by a distinctly decreased FOX leading presumably to a replenishment of the depleted fat depots. This is in line with a suppressed fat oxidation early during RFD of rats after BW loss [50]. In contrast, COX adjusted for BW and FI was higher in R than in C pigs, irrespective of BiW. This might reflect an enhanced utilization of glucose for fat synthesis [16, 48, 49]. A low ratio of FOX/COX was reported to be a risk factor for weight regain in post-dieting human subjects which is similar to our observations in R pigs during early RFD [50]. Possibly, this might be additionally fuelled by an increased intestinal absorptive capacity for carbohydrates as a means of adaptation to FR as shown previously in mice [51, 52].

Food restriction resulted in persistently lower absolute and relative weights of abdominal fat depots after 30 days of RFD independent of the pigs’ BiW. This can be explained by increased lipolysis as indicated by the higher plasma NEFA concentrations and FOX. During the RFD period of 30 days, fat depots were supposedly replenished, but the FR-induced decrease in abdominal fat depot weight still persisted. Whether a previous FR period is still reflected in reduced body fat later on depends on the duration of time and severity of FR, as well as the duration of and energy intake during RFD as shown previously in pigs [30]. Thus, referring to our outgoing hypothesis, the RFD period following the FR period was not long enough to know whether FR leads to a faster rate of fat recovery relative to lean tissue recovery in the longer term.

### Substrate oxidation pattern during 24 h and plasma NEFA concentrations

That in pigs carbohydrates are the major fuel is in line with earlier findings in adequately nourished growing pigs (40–100 kg) where COX provided 85 % of EE, followed by protein oxidation (15 %) with no contribution of FOX to EE in these pigs [53]. This is related to the fact that the typical pig diet is high in carbohydrates and low in fat and thus leads to fat synthesis rather than to FOX [54]. In growing pigs, fat is oxidized if energy supply is insufficient for growth, even if dietary fat content is moderately high [53]. However, in NC pigs with <40 kg BW, we found a small contribution of FOX (8–10 % of total EE), while COX contribution was 80 %. Others reported contributions of 30 % FOX and 55 % COX to total EE in even lighter pigs (30 kg BW) [55]. This confirms that the nutrient oxidation pattern shifts with age in growing pigs toward an increasingly smaller contribution of FOX, and in turn, a higher fat deposition [55, 56].

In food restricted mice (70 % of ad libitum intake), consuming their food in one single meal, FOX did not occur for the next 7 h before it increased and remained at a plateau until the next morning [16]. Compared to the latter study, in our R pigs, the morning FI was insufficient to completely suppress FOX. This is due to the relatively lower amount of dietary energy supplied during the morning meal in our study, leaving the pigs in an energy deficient state. This interpretation is in line with the course of the plasma NEFA concentrations which started to re-increase 4–5 h after the intake of the morning meal and were higher than in C pigs. After 4 days of FR (T2a) in LR pigs, fat balance was neutral between 1.5 and 9 h after the afternoon meal, before FOX re-increased. In contrast, in age-matched NR (T2a) pigs, FOX increased already 6 h after the afternoon meal. This suggests a different diurnal substrate oxidation pattern between L pigs (IUGR) and normal BiW pigs during FR.

## Conclusion

We confirmed that IUGR pigs have a lower capacity for growth and muscle development. Based on our outgoing hypotheses, we observed no differences in EE and RMR between IUGR pigs and their age-matched controls in spite of a higher BW-related FI in IUGR pigs. However, IUGR pigs oxidized less fat than N pigs which underlies their higher body fatness and results in an enhanced fat deposition at an earlier age. During a 3-weeks period of FR, BW gain was delayed, body fat was mobilized and FOX was higher than in ad libitum fed pigs. Food restricted N and L pigs oxidized about 2 times as much fat as their ad libitum fed counterparts. During FR, in LR pigs, FOX was only 50 % of that in NR pigs suggesting a fairly neutral fat balance during FR in IUGR pigs. IUGR pigs showed a different diurnal substrate oxidation pattern than normal BiW pigs during FR. During the first few d of RFD, FI was higher leading to a more positive EB and a more intense stimulation of fat synthesis. This was not different between IUGR and normal BiW pigs. After 30 days of RFD, no differences in components of EE among the four groups remained, but low BiW and FR resulted in higher and lower body fatness of pigs, respectively. Taken together, higher body fat in IUGR pigs, especially abdominal fat, is likely due to a lower potential for muscle accretion, an earlier onset of fat deposition due to a lower ratio of FOX to COX, and a different diurnal fuel selection during FR.

## Electronic supplementary material

Below is the link to the electronic supplementary material.

Supplementary material 1 (DOC 150 kb)
