# Supplementary material for: Higher body fatness in intrauterine growth retarded juvenile pigs is associated with lower fat and higher carbohydrate oxidation during ad libitum and restricted feeding
Source: Eur J Nutr. 2013 Aug 2;53(2):583–97. doi: 10.1007/s00394-013-0567-x (PMC3925302; doi:10.1007/s00394-013-0567-x)
Supplement: Supplementary file 1 — Supplementary material 1 (DOC 150 kb) [file 394_2013_567_MOESM1_ESM.doc]

**Online Resource 1** Dry matter (DM), crude nutrient composition, and metabolizable energy content (ME) in foods and food components

|  | Starter food^1^ | Grower food^2^ | Oat flakes^3^ | Sucrose^4^ |
| --- | --- | --- | --- | --- |
| DM (%) | 98 | 98 | 97 | 98 |
| Crude nutrients (g/kg) |  |  |  |  |
| Crude ash | 58 | 57 | 21 | - |
| Crude protein | 228 | 197 | 143 | - |
| Crude fat | 141 | 32 | 78 | - |
| Carbohydrates | 408 | 545 | 658 | 980 |
| Crude fibre | 21 | 51 | 18 | - |
| Starch | 242 | 447 | 643 | - |
| Sugar | 154 | 57 | 16 | 980 |
| ME (MJ/kg DM) | 17.7 | 14.7 | 17.2 | 14.7 |

^1^ ‘Baby Crisb’, Bergophor, Hohburg Mineralfutter GmbH, Hohburg, Germany

^2^ ‘Vormast CaFo TOP’, Trede und von Pein GmbH, Itzehoe, Germany

^3^ Holstenmühle W. Smidt & Co. KG, Lübeck, Germany

^4^ Nordzucker, Braunschweig, Germany

Groups

NR, LR

NC, LC

***a d l i b i t u m***

**FR**

***ad libitum***

T1 T2a T2b T3a T3b

**RFD**

Age (d) 28 76 83 97 104 125 129

**Online Resource 2** Experimental schedule: Food restriction (FR) and refeeding (RFD) periods and timing of indirect calorimetry measurements; N = normal birth weight, L = low birth weight, C = control/ *ad libitum* feeding, R = restricted feeding.

prior to FR

**Online Resource 3** Daily food intake in pigs with normal (N) and low (L) birth weight starting at weaning, and before and during food restriction (FR; R) and refeeding, compared to permanently *ad libitum* fed age-matched pigs (NC, NR, LC and LR; n = 10 to 12/group). For the sake of clarity error bars were omitted. The 3-wk FR period is shaded in grey.

prior to FR

**Online Resource 4** Body weight development in pigs with normal (N) and low (L) birth weight before and during food restriction (FR; R) and refeeding compared to permanently *ad libitum* fed age-matched pigs (NC, NR, LC and LR; n = 10 to 12/group). For the sake of clarity error bars were omitted. The 3-wk FR period is shaded in grey.

**Online Resource 5** *P*-values of the main factors birth weight class (BiW), feeding type (Feeding), time point (Time) and interactions for various components of energy expenditure (see Table 3)1-3

|  | BiW | Feeding | Time | BiW x Feeding | BiW x Time | Feeding x Time |
| --- | --- | --- | --- | --- | --- | --- |
| BW (kg) | <0.0001 | 0.0193 | <0.0001 | 0.688 | 0.334 | <0.0001 |
| EI^4^ (kJ/kg BW^0.62^ ∙ d^-1^) | 0.298 | 0.040 | <0.0001 | 0.742 | 0.508 | <0.0001 |
| EE^4^ (kJ/kg BW^0.62^ ∙ d^-1^) | 0.909 | 0.006 | <0.0001 | 0.696 | 0.254 | <0.0001 |
| EB^4^ (kJ/kg BW^0.62^ ∙ d^-1^) | 0.307 | 0.366 | <0.0001 | 0.562 | 0.489 | <0.0001 |
| RMR^4^  (kJ/kg BW^0.62^ ∙ d^-1^) | 0.736 | <0.001 | <0.0001 | 0.844 | 0.141 | <0.0001 |
| RQ^4^ | 0.006 | 0.355 | <0.0001 | 0.255 | 0.351 | <0.0001 |
| Q-value (MJ EE/MJ EI) | 0.479 | 0.010 | 0.023 | 0.592 | 0.495 | 0.035 |
| FOX^4^ (g/kg BW^0.62^ ∙ d^-1^) | 0.006 | 0.562 | <0.0001 | 0.183 | 0.283 | <0.0001 |
| COX^4^ (g/kg BW^0.62^ ∙ d^-1^) | 0.026 | 0.842 | <0.0001 | 0.291 | 0.806 | <0.0001 |
| COX^4^  (g/kg BW^0.62^ ∙ kg FI^-1^ ∙ d^-1^) | <0.001 | 0.008 | <0.0001 | 0.466 | 0.247 | 0.304 |

^1^ *Ad libitum* feeding throughout the experiment vs. 3-wk food restriction (between age 80 d and 100 d)

^2^ Time of respiration measurements: age of 76 d, 83 d, 97 d, 104 d, and 125 d

^3^ Three-way interaction BiW x Feeding x Time was not significant for any of the variables (all *P* ≥ 0.2)

^4^ EI, energy intake; EE, energy expenditure; EB, energy balance; RMR, resting metabolic rate; RQ, Respiratory quotient; FOX, fat oxidation; COX, carbohydrate oxidation

**Online Resource 6** Course of plasma NEFA concentrations (µmol/L) within day at d 21 of food restriction (FR; R pigs) and d 7 and 21 of refeeding (RFD) in pigs with normal (N) and low (L) birth weight (BiW) compared to *ad libitum* fed control (C) pigs (see Fig. 3 and 4)^1^

| Time^2,3^ | d 21 of FR^3^  (equivalent to T2b) | | | | | | d 7 of RFD  (equivalent to T3a) | | | d 21 of RFD  (equivalent to T3b) | | | |
| --- | --- | --- | --- | --- | --- | --- | --- | --- | --- | --- | --- | --- | --- |
| (h) | NC | | NR | LC | LR | NR | | LR | NC | | NR | LC | LR |
| -0.1 | 276^Aa^ | | 374^Cab^ | 284^Aa^ | 429^Db^ | 248^B^ | | 270^B^ | 435^B^ | | 426^B^ | 480^B^ | 433^B^ |
| 1 | 47^B^ | | 56^A^ | 52^B^ | 50^A^ | 68^A^ | | 56^A^ | 58^A^ | | 49^A^ | 51^A^ | 41^A^ |
| 2 | 68^B^ | | 62^A^ | 66^B^ | 61^A^ | 55^A^ | | 58^A^ | 59^A^ | | 55^A^ | 74^A^ | 69^A^ |
| 3 | 52^B^ | | 54^A^ | 70^B^ | 59^A^ | 53^A^ | | 69^A^ | 58^A^ | | 54^A^ | 76^A^ | 59^A^ |
| 4 | 47^B^ | | 75^A^ | 59^B^ | 78^A^ | 38^A^ | | 47^A^ | 54^A^ | | 56^A^ | 67^A^ | 66^A^ |
| 5 | 49^B^ | | 96^A^ | 55^B^ | 123^AB^ | 47^A^ | | 46^A^ | 60^A^ | | 49^A^ | 57^A^ | 73^A^ |
| 6 | 53^Ba^ | | 157^ABab^ | 51^Ba^ | 199^BCb^ | 49^A^ | | 56^A^ | 54^A^ | | 50^A^ | 49^A^ | 57^A^ |
| 7 | 51^Ba^ | | 251^Bb^ | 68^Ba^ | 246^Cb^ | 51^A^ | | 53^A^ | 48^A^ | | 55^A^ | 50^A^ | 57^A^ |
| 8 | 51^B^ | | 79^A^ | 56^B^ | 61^A^ | 48^A^ | | 76^A^ | 59^A^ | | 51^A^ | 47^A^ | 56^A^ |
| 12 | 59^B^ | | 110^A^ | 60^B^ | 100^AB^ | 51^A^ | | 49^A^ | 53^A^ | | 50^A^ | 50^A^ | 50^A^ |
| *P*-values^4^ | | | | | | | | | | | | | |
| BiW | | 0.365 | | | | | 0.298 | | | 0.460 | | | |
| Feeding Type | | <0.0001 | | | | | - | | | 0.625 | | | |
| Time | | <0.0001 | | | | | <0.0001 | | | <0.0001 | | | |

^A-C^ Within a column, LSM without a common capital superscript differ between the time points within one group (*P* ≤ 0.05; Tukey-Kramer test)

^ab^ Within a row, LSM without a common lowercase superscript differ within one time point on d 21 of FR (*P* ≤ 0.05; Tukey-Kramer test)

^1^ Values are LSM (n = 6 to 9/ group); pooled SE: T2b, 22.3; T3a, 14.7; T3b, 21.8.

^2^ Time is expressed relative to the morning meal.

^3^ The afternoon meal was given immediately after the 7 h blood sample.

^4^ Feeding x Time, BiW x Feeding, BiW x Time, and BiW x Feeding x Time interactions were not significant (*P* > 0.9), with the exception of the interaction Feeding x Time at time point T2b (*P* < 0.0001).
